# Robotic-guided radiofrequency ablative disconnection of hypothalamic hamartoma

**DOI:** 10.3171/2024.4.FOCVID2450

**Published:** 2024-07-01

**Authors:** Ramesh Sharanappa Doddamani, Poodipedi Sarat Chandra, Ravichandra Kota, Nihal Ahemad, Manjari Tripathi

**Affiliations:** Departments of 1 Neurosurgery and; 2 Neurology, All India Institute of Medical Sciences, New Delhi, India

**Keywords:** radiofrequency ablation, epilepsy, gelastic seizures, ROSA

## Abstract

Hypothalamic hamartomas (HHs) are benign masses, often associated with drug-refractory epilepsy (DRE). Open surgery as well as the endoscopic disconnection techniques are fraught with a high risk of morbidity and failure rates. The authors have been performing robotic-guided radiofrequency (RF) ablation for all types of HH presenting with DRE as a standard procedure at their institution. The authors have operated on 25 patients with HH using this technique over the last 8 years. This is a safe, effective, and minimally invasive technique. In this video article, the authors intend to demonstrate their technique of RF ablative disconnection under robotic guidance.

The video can be found here: https://stream.cadmore.media/r10.3171/2024.4.FOCVID2450

## Transcript

In this video, we present our technique of robotic-guided radiofrequency ablation for hypothalamic hamartoma presenting with drug-refractory epilepsy.

### 0:28

A 15-year-old boy presented with history of gelastic epilepsy beginning from the age of 5 years with 3–4 episodes of gelastic seizures per day and were refractory to antiseizure medications. Over the last 5 years, the boy has developed new seizure types in the form of head drops and generalized seizures.

Examination was normal except for mild refractory error in the left eye.

There was no significant perinatal history, and he was on multiple antiseizure medications.

### 0:57

Video EEG is often nonlocalizing in these cases, as these lesions are deep seated. In this case the video EEG localized to the left frontal region. On neuropsychological assessment, mildly impaired intellectual function was noted.

### 1:10

He was further evaluated with 3T MRI brain acquired in 3D sequences, MPRAGE sequences, T2-weighted image, FLAIR, and double-contrast sequences are routinely performed in these cases. The protocol for these sequences is 3D acquisition of all the sequences with slice thickness of 1 mm in square matrix without any gap. The MRI brain showed noncontrast-enhancing lesion located in the third ventricle projecting into the suprasellar cisternal space, arising from the hypothalamus, pathognomonic of hypothalamic hamartomas. The lesion is also seen to be attached to bilateral hypothalami by broad pedicles representing Delalande’s type III hamartoma. The clinical picture of gelastic epilepsy since childhood along with the classical MRI findings is diagnostic of hypothalamic hamartomas. Hence there is no need for further investigations.

### 1:55

Once the diagnosis is confirmed, surgical management is planned. The concept of disconnection of the hamartomas was first described by Delalande and collegues.^1^ The surgical treatment of hamartomas has undergone a sea change, beginning from the era of open skull base approaches for resection of these lesions, to endoscopic transventricular disconnection, to the current standard of minimally invasive ablative disconnection either using radiofrequency or laser ablations.^2–8^ At our institution we have been performing robotic-guided radiofrequency ablation for these lesions as a standard treatment since the last 8 years.^9,10^

### 2:27 Trajectory Planning.

The disconnection is planned along the interface between the normal hypothalamus and the hamartoma. The planning of the trajectories is performed a day prior to the surgery. The pedicle dimensions are noted in 3D, that is, in the superomedial to inferomedial planes bilaterally, as well as in the anteroposterior plane.

### 2:48

The 3D T1 MPRAGE sequence is preferred for clear identification of the interface between the hamartoma and the normal hypothalamus necessary for accurate planning. A double-contrast MRI is extremely useful to avoid the vascular structures, while planning the trajectories. We use a radiofrequency probe with an active tip of 4-mm length and 2.2-mm diameter, which produces a cylindrical lesion of approximately 6-mm diameter and a length of 4–5 mm. Hence, the first lesion is planned so that 5-mm length of the probe tip should lie within the hamartoma from the interface. We proceed with lesioning in a centripetal manner, especially when contralateral pedicle is to be lesioned. Depending upon the length of the contralateral attachment, further lesions are spaced 4 mm along the depth. Each trajectory is planned in 3D sequence and spaced 6 mm apart in such a way that there exists an overlap but without any skip lesions.

### 3:43

The hypothalamic hamartoma is surrounded by critical neurovascular structures: bilateral hypothalami forming the lateral boundaries, the optic nerves and the tract occupying the anterolateral aspect of the hamartoma, the anterior cerebral arteries in the anterolateral aspect bilaterally, the basilar artery in the midline posterior aspect, and lastly the fornix and the anterior commissure occupying the anterior and superior aspect of the hamartoma. Hence, while planning the trajectories, the target points should be planned within the hamartoma avoiding transgression of the optic nerve and the fornices, while maintaining a safe distance from the blood vessels.

### 4:18

We planned 8 trajectories, 4 on each side. A total of 12 lesions were created, 8 on the left side while 4 near the right-sided attachment at the interface. The lesioning is performed from outside-to-inside technique by advancing the probe by 4 mm each time. All the trajectories were drilled from the right side only.

### 4:41 Operating Room Setup.

 We use ROSA robot, O-arm, Leksell pulse generator, adaptors for ROSA arm of different sizes, radiofrequency probes, hand gun for fashioning the twist drill with drill bits of 2.5 mm in diameter, and a monopolar coagulator.

### 5:00 Patient Positioning.

The patient is positioned supine, head is fixed with the Mayfield clamp, and attached to the robotic arm to achieve rigid fixation. We routinely use skin-based laser registration which begins initially with a 6-point facial registration. This is followed by the mesh registration covering the nose and the forehead region, followed by zigzag laser scanning of the forehead and then subsequently manual scanning of the bilateral temple regions.

### 5:44

After the registration is complete, the accuracy is checked by pointing the laser to all the 6 points. The aim here is to attain a minimum error. If the error is more than 1 mm, then the points can be manually adjusted to correct it. In case of persistence of the error re-registration should be done.

### 6:11

The robotic arm is moved to the desired trajectory and the entry point is marked as per the laser pointer.

### 6:19 Surgical Technique.

 The robotic adapter should be closely opposed to the scalp in order to avoid play of the drill bit, thereby preventing gross deviations. While drilling, the surgeon can very well appreciate resistance initially representing the outer cortical bone, followed by a give-away feeling denoting entry into the diploic space. This is again followed by a resistance suggestive of inner cortical margin, and a final give-away suggesting entry into the epidural space. This is followed by dural coagulation using a monopolar cautery.

### 6:50

The distance to the target is displayed on the robotic screen, which represents distance from the top of the adapter to 5 mm deeper to the hamartoma interface. This length is marked on the radiofrequency coagulation probe using a scale.

### 7:09

Once the probe is placed at the desired length, an O-arm spin is performed which is merged with the preoperative MRI to note the accuracy. This process is performed for the first trajectory, before proceeding to the lesioning, to confirm the accuracy.

### 7:22

The lesions are performed as per the preoperative planning, at 74° and 60 seconds.

### 7:28

Prevention of the CSF leak is of utmost importance during withdrawal of the probe, which if unchecked can lead to significant brain shift and thereby significantly diminish the accuracy of the burn lesion. This can be tackled by injecting the fibrin glue and thereby plugging the dural defect.

### 7:44

Postoperative imaging shows good lesioning at the interface bilaterally. Postoperative course was uneventful, without any neurological deficits, and hormonal workup was within normal limits.

### 7:56

Patient is seizure free at 1-year follow-up with ILAE class IA outcome. These are my references. Thank you.

## Acknowledgments

We thank Gaurav Rawat, Technical Assistant, for dedicatedly operating the robot during surgery (ROSA).

## Disclosures

The authors report no conflict of interest concerning the materials or methods used in this study or the findings specified in this publication.

## Author Contributions

Primary surgeon: Chandra, Doddamani. Assistant surgeon: Kota, Ahemad. Editing and drafting the video and abstract: Chandra, Doddamani, Ahemad, Tripathi. Critically revising the work: Chandra, Doddamani, Tripathi. Reviewed submitted version of the work: Chandra, Doddamani, Kota, Tripathi. Approved the final version of the work on behalf of all authors: Chandra. Supervision: Chandra.

## Supplemental Information

### Patient Informed Consent

The necessary patient informed consent was obtained in this study.
